# Can differences in medical drug compliance between European countries be explained by social factors: analyses based on data from the European Social Survey, round 2

**DOI:** 10.1186/1471-2458-9-145

**Published:** 2009-05-16

**Authors:** John Larsen, Henrik Stovring, Jakob Kragstrup, Dorte G Hansen

**Affiliations:** 1 Research Unit of General Practice, Institute of Public Health, University of Southern Denmark, Odense, Denmark

## Abstract

**Background:**

Non-compliance with medication is a major health problem. Cultural differences may explain different compliance patterns. The size of the compliance burden and the impact of socio-demographic and socio-economic status within and across countries in Europe have, however, never been analysed in one survey. The aim of this study was to analyse 1) medical drug compliance in different European countries with respect to socio-demographic and socio-economic factors, and to examine 2) whether cross-national differences could be explained by these factors.

**Methods:**

A multi-country interview survey *European Social Survey, Round 2 *was conducted in 2004/05 comprising questions about compliance with last prescribed drug. Non-compliance was classified as primary and secondary, depending whether the drug was purchased or not. Statistical weighting allowed for adjustment for national differences in sample mechanisms. A multiple imputation strategy was used to compensate for missing values. The analytical approach included multivariate and multilevel analyses.

**Results:**

The survey comprised 45,678 participants. Response rate was 62.5% (range 43.6–79.1%). Reported compliance was generally high (82%) but the pattern of non-compliance showed large variation between countries. Some 3.2% did not purchase the most recently prescribed medicine, and 13.6% did not take the medicine as prescribed. Multiple regression analyses showed that each variable had very different and in some cases opposite impact on compliance within countries. The multilevel analysis showed that the variation between countries did not change significantly when adjusted for increasing numbers of covariates.

**Conclusion:**

Reported compliance was generally high but showed wide variation between countries. Cross-national differences could, however, not be explained by the socio-demographic and socio-economic variables measured.

## Background

Non-compliance with medication is a major health problem worldwide and prevalent for all kinds of drugs and degrees of diseases. Poor compliance may have a far greater impact on the health of the population than any improvement in specific medical treatments [1]. Further, compliance differs substantially across countries [2,3]. Usually, the limit between good and poor compliance is set at 80% [4]. Whether or not advice about drug treatment is followed depends on various factors including physician, patient, communicative, social and cultural circumstances. European societies are highly diverse with regard to family structures, education, employment practices, ethnicity, religious beliefs, and also the structure of healthcare systems. Hence, wide variation between countries in attitudes towards drug taking may be expected, i.e. patient behaviour concerning compliance with prescribed drugs may vary between countries due to cultural differences [2,3]. The impact of socio-economic status has been divergently reported [5-9]. However, diverging outcomes may be explained by methodological or contextual differences. This paper is based on the first large-scale multi-country survey including information on drug use and compliance as well as socio-demographic and socio-economic factors.

The aim of the study was by way of a multi-level approach to analyse 1) medical drug compliance in European countries with respect to socio-demographic and socio-economic factors and to examine 2) whether cross-national differences could be explained by socio-demographic and socio-economic factors (citizenship, ethnic status, sex, age, education, profession, household income).

## Methods

We conducted a multi-country study based on data from the European Social Survey, ESS round 2, 2004/2005 including 24 countries and comprising 73,090 individuals [10]. According to national options, participants were sampled by means of telephone books, postcode address files, population registers or social security register data. In the sampling procedure it was ensured that regardless of the method used for a specific country, the statistical precision was the same for all countries. In each country information was collected using a main and a supplementary questionnaire filled in through an hour-long face-to-face interview including questions on use of medicine, immigration, citizenship, socio-demographic and socio-economic issues [11]. The ESS questionnaire was translated into the language of each of the participating countries by language experts, supported by the ESS Translation Taskforce and further supervised by a Central Coordinating Team [10].

Data are kept within and distributed by the Norwegian Social Science Data Services (NSD) [12]. Data is openly available at the homepage.

Information on compliance behavior related to question D9 in the questionnaire: *Please think back to the last time a doctor prescribed you a medicine you had not had before. Which statement on the card comes closest to what you did with this prescription? 1: I didn't collect the medicine from the pharmacy; 2: I collected the medicine but didn't use any of it; 3: I used some or all of the medicine but not exactly as prescribed; 4: I used the medicine exactly as prescribed; 5: can't remember last occasion*. Individuals were categorized as compliant (4^th ^statement), primary non-compliant (1^st^) or secondary non-compliant (2^nd ^or 3^rd ^statement). The independent variables included: gender, age, education level (five levels based on level of completed education), household income (twelve groups), cohabitation (living with or without a partner), ethnic minority (belonging or not belonging to an ethnic minority group), and profession. The latter was classified according to the occupation standard ISCO 88, the official classification standard of the International Labour Organization (ILO) [13]. To compare professional groups across European countries, individuals were further categorised into four groups (Controller/Non-manual, Self-employed, Manual, and Farming) according to a slight modification of the model suggested by Leiulfsrud et al for use with the ESS data [14,15].

### Statistical analyses

As not all countries could include subjects with equal probability, the data contains weights which correct for this when included in the analysis. Further, the weights allows for differences in population size between countries, such that even though country specific samples have approximately similar sizes, larger countries contribute more than smaller countries in analyses across countries when weights are used [16]. To account for non-response to individual items, a multiple imputation strategy [17] was applied for all variables studied, i.e. both with the respect to the compliance question and the explanatory variables. Country was included as an additional explanatory variable in imputations. The imputation and subsequent analyses were conducted using the *ice *and *micombine *procedures available in Stata 9.2 [18]. The multiple imputation strategy is valid upon the Missing At Random assumption, i.e., that the values missing can be unbiasedly predicted from the remaining observed data. It should be noted, that using the response variable as a predictor in the model for imputations, does not invalidate subsequent analyses of the same outcome with respect to the covariates of interest [19].

To achieve a measure for household income meaningful across countries we created a log-mean scale for income in different countries, as this was found to yield a reasonable linear relationship with compliance on a log-odds-scale. Consequently, log-income was used as a linear covariate in logistic regression analyses of compliance.

Chi square test followed by Fisher's Exact Test was used to evaluate differences between categories. A multivariate analysis was undertaken to adjust for potential confounding of variables on each other. In order to assess the effect of covariates on the variation of compliance between countries, a multilevel analysis was made. A full-fledged analysis with country-dependent random effects was numerically intractable on available computers – except for the most basic situation with only one country-dependent random effect. Instead, we set up a two-step fixed model based on logistic regression: First, a fixed effect model was fitted via logistic regression for each country independently. Relevant estimates of effects (on the log-odds scale) from these models were then joined together and their standard deviation was computed. Confidence intervals for standard deviations were computed using bootstrap methodology. This was done both with and without relevant covariates, to allow for assessing how much of the observed variation in compliance between countries could be explained by adjusting for observed covariates. Subsequently, we summarized the variation in dependence of covariates between countries. All covariates were centered at their mean to yield fair comparisons of adjusted and unadjusted variations between countries.

Stata 9.2 was used for all analyses.

## Results

The mean response rate was 62.5% varying between 43.6% in France and 79.1% in Estonia. In total 45,678 participants from 24 countries were included ranging from 579 in Iceland to 3,026 in the Czech Republic. A total of 41,102 participants answered the compliance questions. The 45,678 participants represented an effective (i.e. weighted) sample size of 37,718. Additional file 1 shows the distribution of the socio-demographic and socio-economic variables within countries weighted with respect to design and sampling differences. (Additional file 2 further includes 95% confidence intervals for all parameters).

**Figure 1:**
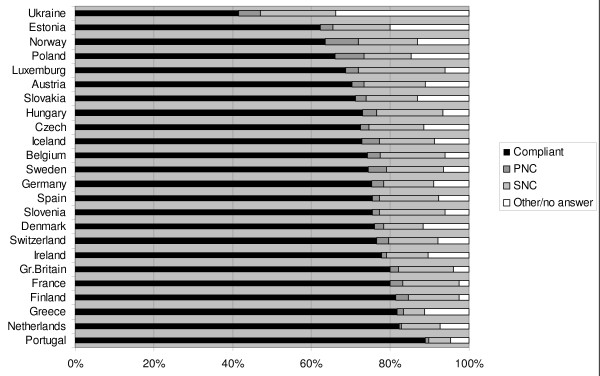
**Total compliance and primary and secondary non-compliance in 24 European countries**.

The pattern of reported compliance showed large variation (Figure 1). Across Europe, non-compliance with latest prescribed drug was 16.8%, varying from 6.4% in Portugal to 24.9% in Luxemburg. Some 3.2% did not collect the medicine at all (primary non-compliance) (0.6% in the Netherlands to 8.5% in Norway), while 13.6% (5.6% in Portugal to 21.6% in Luxemburg) did not take the medicine as prescribed (secondary non-compliance). 7.1% did not remember last occasion, 1.4% never had a drug prescribed, and 1.9% answered otherwise or did not answer at all.

Additional file 3 shows the degree of reported compliance according to socio-demographic and socio-economic categories. The multiple regression analysis including gender, age, cohabitation, education, income, profession, ethnic minority, and country (additional file 4) showed no difference in reported compliance between males and females but compliance increased with age. People living with a partner were significantly more compliant than singles. People with an education longer than 12 years were significantly less compliant than those with shorter education but people living in households with higher incomes were significantly more compliant. Manual workers and ethnic minority groups were significantly less compliant than the other groups. The effect of each variable varied substantially between the countries. For example, the female:male OR ranged from 0.86 to 1.52.

In the multilevel analysis the variation between countries was expressed as the standard deviation (SD) i) without correction, ii) with correction for gender and age, and iii) with correction for all covariates. The SDs were 0.39 (CI95% 0.25–0.54), 0.39 (0.25–0.54) and 0.42 (0.23–0.62). Similar numbers for more homogenous subgroups of respondents are shown in additional file 5. Thus, the variation in compliance between countries did not change significantly even when adjusting for increasing numbers of covariates.

## Discussion

A relatively high level of reported compliance was observed, but with substantial variation between the 24 European countries. The total, primary and secondary non-compliance differed as did the association with socio-demographic and socio-economic factors. Across boundaries, however, no general association with these social factors was found.

The questionnaire of round 2 of the European survey – in contrast to round 1 -included questions specifically designed to illustrate compliance issues. Although the questions were not specifically designed for the present study, we believe that they suffice to satisfy our compliance definitions.

In the design phase of the survey, considerable effort was used to minimise sampling bias to ensure that the obtained sample – when weighted appropriately – was representative of the source population studied. As the questionnaire was an omnibus covering many different aspects of life, non-participation cannot be directly related to a specific subject area such as non-compliance, although a subtle relation may exist due to underlying factors. In the analysis phase we further employed a multiple imputation strategy to include subjects with partially missing responses, and thus we have increased statistical efficiency as no subjects have been excluded due to partial non-response. The generally high compliance reported may be an overestimate due to other kinds of bias, e.g. recall or "honesty" bias, i.e. participants may have been reluctant to report non-compliance. The willingness to answer truthfully may further differ across countries. On the other hand, compliance may have been underestimated due to the rather strict definition: "I used the medicine exactly as prescribed". The definition was furthermore based on the most recently prescribed drug they "had not had before". This has most likely been a drug for an acute condition, with which compliance is known to be better than with chronic medication [20]. We have, however, no reason to believe that this invalidates our conclusions regarding between country comparisons.

Analyses of variables based on a large number of categories gathered in substantially different countries require collapse of categories. Working with the ISCO 88 occupation standard we found a class scheme with four groups appropriate. More categories would have made the model infeasible as one or more would not have been represented in all countries. Fewer categories, on the other hand, would have diminished the value of the variable. Using a log scale for household income instead of the actual figures made it possible to compare the impact of income in different countries.

Compliance depends on a variety of factors including indication, the drug, side effects, and drug regimen etc [21]. Compliance with short antibiotic regimens has been reported to approach 100% [22], whereas long-term compliance with chronic medication has been reported to be as low as 50% [23]. A Swedish study reported primary non-compliance to be 2.4% but with three-fold variation depending on substance [24]. We are not aware of any other studies comparing compliance in a large number of culturally diverse countries. However, cross-national studies comparing compliance in two areas/countries have been published [2,4] with observations of substantial differences in compliance between regions. These studies are methodologically different as they are based on prescription claims data.

Across countries we found that compliance increased with age, which may be due to the fact, that younger people are less authoritarian and do not feel the same burden of sickness. They are therefore not apt to follow medication as strictly as older people. More elderly may, however be affected by chronic diseases and be prone to polypharmacy known to decrease compliance [25,26]. Across countries there may be unrecovered differences in the pattern of chronic diseases or polypharmacy. Living with a partner meant better compliance, perhaps because of a more structured daily routine than among those living alone. People with the shortest education were more compliant than those with a longer one, suggesting that the better educated people feel more responsible for their own health and make decisions about discontinuing drug treatment [27]. In contrast, higher income was associated with better compliance, reflecting the simple fact that wealthier people can better afford the drugs. However unexplainably, manual workers seem less compliant than more occupationally independent groups. Underuse of prescribed drugs may be cost-related [28,29]. However, in this survey we did not have information on drug cost or specific reimbursement rules in the participating countries.

## Conclusion

In conclusion, compliance was generally reported to be high. There was a wide variation between countries. The differences could not be explained by the socio-demographic and socio-economic variables investigated in this study. Thus, other factors must contribute to the variation to a large extent, e.g. differences in the availability of medications between countries, additional factors, differences in the prevalence of specific diseases or conditions, health insurance systems, etc. This is a natural issue for further research.

## Competing interests

The authors declare that they have no competing interests.

## Authors' contributions

JL participated in the design, coordination, and sequence alignment of the study and drafted the manuscript. HS participated in the design of the study and performed the statistical analysis. JK conceived of the study and participated in the design of the study. DGH participated in the design of the study and in the sequence alignment. All authors read and approved the final manuscript.

## Pre-publication history

The pre-publication history for this paper can be accessed here:



## Supplementary Material

Additional File 1 **The European Social Survey, Round 2, 2004/5. Participants (weighted counts) by total N and percentages distributed by country and socio-demographic and socio-economic variables (95% confidence intervals shown in additional file **2). This file shows the distribution of the socio-demographic and socio-economic variables within countries weighted with respect to design and sampling differences. Click here for file

Additional File 2 **The European Social Survey, round 2, 2004/5. Participants (weighted counts) by total N and percentages including 95% confidence intervals distributed by country and socio-demographic and socio-economic variables**. In addition to the data presented in the additional file 1, this file further includes 95% confidence intervals for all parameters. Click here for file

Additional File 3 **Level of compliance according to categories (percentages, odds ratios and 95% confidence intervals) in the European Social Survey, round 2, 2004/5**. This file shows the degree of reported compliance according to socio-demographic and socio-economic categories. Click here for file

Additional File 4 **Odds ratios for compliance adjusted for all covariates including country (country of residence omitted in the table) in the European Social Survey, round 2, 2004/5**. *Log odds for trend (income on log-scale with base 2). This file represents the multiple regression analysis of compliance including gender, age, cohabitation, education, income, profession, ethnic minority, and country. Click here for file

Additional File 5 **The variation between countries expressed as standard deviation (SD (95% CI)) for all and for more homogenous subgroups of respondents**. This file represents the analysis of the variation of compliance between countries, unadjusted as well as adjusted for various and all covariates. Click here for file
